# Cardiac Surgery During Pregnancy

**DOI:** 10.1016/j.atssr.2022.08.004

**Published:** 2022-08-19

**Authors:** Elizabeth H. Stephens, Joseph A. Dearani, William Mauermann, Ellen M. Bendel-Stenzel, Katherine W. Arendt, Carl H. Rose, Caitlin Blau, Juan Crestanello, Hartzell Schaff

**Affiliations:** 1 Department of Cardiovascular Surgery, Mayo Clinic, Rochester, Minnesota; 2 Department of Anesthesiology, Mayo Clinic, Rochester, Minnesota; 3 Department of Neonatology, Mayo Clinic, Rochester, Minnesota; 4 Department of Maternal-Fetal Medicine, Mayo Clinic, Rochester, Minnesota; 5 Department of Cardiovascular Perfusion, Mayo Clinic, Rochester, Minnesota

## Abstract

The need for cardiovascular surgery during pregnancy is infrequent but is expected to increase as more patients with congenital heart disease live into childbearing years. Care for women considering pregnancy with residual, recurrent, or newly identified lesions related to congenital heart disease disease must address therapeutic options that maximize the mother’s health while acknowledging concerns for procedural effects on fetal development and risks associated with preterm delivery. This report summarizes the current knowledge regarding optimal intraoperative techniques for the pregnant patient and provides recommendations to optimize maternal and fetal outcomes.

Cardiac surgery during pregnancy occurs infrequently but remains necessary in cases where maternal well-being is at risk. Preconception counseling with anticipatory management of maternal cardiac disease is ideal, but unplanned pregnancy and unexpected maternal decompensation can lead to situations in which surgery cannot be deferred. The goal of this report is to detail procedural differences during intrapartum cardiac surgery for the cardiac surgical team to optimize maternal and fetal outcomes.

Pregnancy in a woman with cardiac disease should prompt discussion among the pregnancy heart team members (cardiology, maternal-fetal medicine, cardiac surgery, neonatology, and anesthesia specialists)^1^ regarding the *necessity* of cardiac surgery during pregnancy and the *timing* of surgery relative to the gestational age of the fetus. Risk mitigation favors percutaneous procedures (eg, for valve stenosis or bioprosthetic valve dysfunction and aortic [re]coarctation) and avoidance of surgery at an early gestational age to minimize fetal complications, with consideration of preterm delivery followed by cardiac surgery.^2^^,^^3^

## Technique

Cardiac surgery during pregnancy requires thoughtful planning and a well-orchestrated team. Key principles include minimizing bypass and clamp time as well as reducing blood loss. Indications for emergency delivery should be clearly defined.

### General Considerations and Fetal Care

Before cardiac surgery, maternal corticosteroids should be administered for gestational age 23 to 34 weeks to optimize fetal outcomes in the event of delivery. Maternal positioning is with left lateral uterine displacement (wedge under maternal right hip) to reduce the hypotensive effects of aortocaval compression at gestational age greater than 20 weeks. Sequential lower extremity compression devices reduce the risk of venous thrombosis. Medications should be reviewed and deemed safe for pregnancy; penicillin and cephalosporin antibiotics are acceptable. Nonsteroidal analgesics are avoided because of the possibility of ductal constriction, with the effect more pronounced at ≥32 weeks of gestational age.

Optimizing maternal oxygen saturation (ideally >95%), confirming adequate ventilation, and avoiding maternal alkalosis, acidosis, hypoglycemia, and hypothermia are key because the latter conditions are associated with fetal distress.^4^ Hyperoxia, hypoxia, hypercarbia, and hypocarbia, as well as acid-base status, all affect uteroplacental blood flow and oxygen transport to the fetus. Pco_2_ goals are 30 to 35 mm Hg to maintain normal blood gases for pregnancy, as follows: pH, 7.40 to 7.47; Po_2_, 105 to 110 mm Hg; Pco_2_, 30 mm Hg; and bicarbonate, 20 mm Hg.

Decisions on intraoperative fetal monitoring and tocodynamometry should take into account fetal viability and maternal preference regarding emergency cesarean delivery for fetal distress.^2^ Normal fetal heart rates range between 110 and 160 beats/min, and flow rate, mean arterial pressure, and maternal temperature are adjusted for fetal heart rate slowing. Fetal heart rate variability becomes minimal to absent under maternal-fetal anesthesia and is no longer a useful indicator to assess for acidosis.

### Operative Considerations

Before bypass, 400 U/kg of heparin is administered with a targeted activated clotting time (ACT) of >480 seconds. For an ACT lower than target, an additional 50 U/kg of heparin is administered, and ACT is rechecked. The bypass circuit prime includes Plasma-Lyte (Baxter Healthcare Corp), 0.8 to 1.5 L. If the calculated anticipated hematocrit is less than 24%, red blood cells are added to the prime. Circuit volume is minimized with retrograde arterial or venous autologous priming.

Normothermic, high-flow cardiopulmonary bypass is used. Cardiac output is elevated during pregnancy, and high flow optimizes uteroplacental circulation; hypothermia is associated with fetal bradycardia and premature labor. Flow is greater than 3.0 L/min/m^2^ while maintaining a mean arterial pressure of greater than 70 mm Hg.^3^ The average normal hemoglobin during pregnancy is 11.7 g/dL, and a similar level (>10 g/dL) is maintained. Secondary markers of adequate perfusion include an oxygen delivery index of >300 mL O_2_/min/m^2^. When the mean arterial pressure drops to less than 70 mm Hg, vasoconstrictors such as ephedrine or vasopressin are used, with emphasis on increasing flow and thereby placental perfusion. Although ephedrine can be used in moderation, it crosses the placenta and can increase fetal metabolic rate; vasopressin is often avoided in pregnancy because of a lack of evidence on its effect on the uteroplacental unit, as well as its structural similarity to oxytocin. Phenylephrine is avoided because it crosses the placenta and can cause placental vasoconstriction. Antifibrinolytic agents such as tranexamic acid are avoided because pregnancy is an intrinsically hypercoagulable state; the use of these agents should be confined to those patients with bleeding concerns.

In cases when cesarean delivery occurs immediately before cardiac surgery, delivery is performed with the patient under general anesthesia. After delivery, the uterine incision is closed, and the abdominal wound is packed. Uterotonic agents should be used with caution given their potential effects on systemic and pulmonary vascular tone, although these effects may be obviated by bypass and vasoactive medications. At the time of cord clamping, 1 g of intravenous tranexamic acid is administered and repeated after 30 minutes. Oxytocin is an effective uterotonic agent but causes a dose-dependent decrease in systemic vascular resistance; it should be administered starting at a rate of 6 IU/h and increased to 18 IU/h as needed to improve tone while counteracting hypotension with phenylephrine. Rectal or buccal misoprostol, 800 μg, may be used to improve uterine tone with few cardiovascular effects. Note that methylergonovine acts similarly to an α-agonist and causes vasoconstriction, and carboprost is a prostaglandin F_2α_ agonist and can cause significant increases in pulmonary vascular resistance.

The patient is then positioned supine, median (re)sternotomy is performed, and cardiac surgery proceeds in routine fashion, excluding the use of tranexamic acid. Nitroglycerin and sodium nitroprusside can cause uterine relaxation with resultant obstetric hemorrhage in combination with heparinization and should be avoided. A member of the obstetric team should remain present to assess fundal height and uterine tone and to intervene on uterine bleeding as needed. In very rare cases (eg, critical aortic stenosis), sternotomy is recommended before cesarean delivery in the event that bypass is needed for maternal instability during delivery. After the chest is closed, the abdominal wound is then reopened, hemostasis is secured, and the incision is closed. There is usually 500 to 1000 mL of blood loss from the cesarean delivery to be considered in maintaining the patient’s hematocrit.

## Comment

By using a multidisciplinary approach and making a careful risk-benefit analysis of maternal and fetal health, including shared decision making with the mother, results have been commensurate with expected risk. Published results from our institution (Mayo Clinic, Rochester, MN) of 21 pregnant women who underwent cardiac surgery, including 12 emergency cases, reported 1 perioperative maternal death, in a patient who underwent an emergency mechanical aortic valve thrombectomy. There were 7 combined cesarean-cardiopulmonary bypass procedures (33%), 3 preterm deliveries (21%), and 3 fetal deaths in continuing pregnancies (21%).^5^ A metaanalysis found an 11% rate of maternal death, a 9% incidence of significant maternal complications, and a 33% rate of fetal loss.^6^ These investigators speculated that the discordant rate of adverse results compared with earlier studies could represent a previous selective reporting of favorable outcomes.^6^ Subsequent publications have described a maternal mortality rate of 37% with fetal mortality in 66% of cases of emergency surgical thrombectomy,^7^ as well as combined cesarean-cardiopulmonary bypass in 50% (2 of 4) of patients, with a 50% preterm delivery rate in the continuing pregnancies.^8^
